# Extraction of cage-like sporopollenin exine capsules from dandelion pollen grains

**DOI:** 10.1038/s41598-018-24336-9

**Published:** 2018-04-26

**Authors:** Tengfei Fan, Jae Hyeon Park, Quynh Anh Pham, Ee-Lin Tan, Raghavendra. C. Mundargi, Michael G. Potroz, Haram Jung, Nam-Joon Cho

**Affiliations:** 1 School of Materials Science and Engineering and Centre for Biomimetic Sensor Science, Nanyang Technological University, 50 Nanyang Drive, 637553 Singapore, Singapore; 2 School of Chemical and Biomedical Engineering, Nanyang Technological University, 62 Nanyang Drive, 637459 Singapore, Singapore

## Abstract

Pollen-based microcapsules such as hollow sporopollenin exine capsules (SECs) have emerged as excellent drug delivery and microencapsulation vehicles. To date, SECs have been extracted primarily from a wide range of natural pollen species possessing largely spherical geometries and uniform surface features. Nonetheless, exploring pollen species with more diverse architectural features could lead to new application possibilities. One promising class of candidates is dandelion pollen grains, which possess architecturally intricate, cage-like microstructures composed of robust sporopollenin biopolymers. Here, we report the successful extraction and macromolecular loading of dandelion SECs. Preservation of SEC morphology and successful removal of proteinaceous materials was evaluated using scanning electron microscopy (SEM), matrix-assisted laser desorption/ionization-time of flight (MALDI-TOF) mass spectrometry, elemental CHN analysis, dynamic image particle analysis (DIPA) and confocal laser scanning microscopy (CLSM). Among the tested processing schemes, acidolysis using 85% (v/v) phosphoric acid refluxed at 70 °C for 5 hours yielded an optimal balance of intact particle yield, protein removal, and preservation of cage-like microstructure. For proof-of-concept loading, bovine serum albumin (BSA) was encapsulated within the dandelion SECs with high efficiency (32.23 ± 0.33%). Overall, our findings highlight how hollow microcapsules with diverse architectural features can be readily prepared and utilized from plant-based materials.

## Introduction

Pollen-based microcapsules have attracted much interest as microencapsulation materials due to their eco-friendly nature, uniform micron-scale size, and chemical and physical stability^1,2^. In more detail, the outermost layer, or exine, of the pollen grain wall is significantly composed of sporopollenin – an extremely robust mixture of biopolymers resistant to non-oxidative physical, biological and chemical degradation procedures^3^. On the other hand, the innermost layer, or intine, of pollen grain walls consist of cellulose, pectin, protein and polysaccharides susceptible to acetolysis of conventional processing methodologies^4,5^. As such, the distinct robustness of sporopollenin make hollow sporopollenin exine capsules (SECs) a promising material for applications in drug delivery^6–13^, electronics^14^, and as templates for advanced materials^15^.

Given this, various schemes for the extraction of hollow SECs from different plant spores have been developed, with obtained capsules free from cytoplasmic material, proteins, and the intine layer^3,16^. These schemes often consist of complicated and time-consuming steps involving alkaline lysis, acidolysis, and enzymatic processes. Furthermore, harsh reflux conditions such as high temperature, use of toxic solvents and prolonged heating, are often necessary^16^. This poses a particular problem for more complex or highly ornamented pollen grains, with such aggressive treatment makes it challenging to perform quality extraction of hollow SECs featuring minimal damage to the exine surface. In fact, the majority of SEC extractions developed thus far relate primarily to natural pollen species possessing largely spherical geometries and uniform surface features^3,16^. Only in recent years have more moderate reaction conditions for SEC extraction been investigated^17,18^.

An example of an SEC that would benefit from the development of more moderate extraction protocols is that derived from dandelion pollen grains. Here, the tough outer exine layer possess intricate lophate sculpturing as characterized by a pattern of ridges surrounding depressions forming an intricate cage-like microstructure. Furthermore, the intine layer of dandelion pollen grains is significantly thicker than those found in previously studied pollen grains (1–1.5 μm *vs*. < 700 nm for *Lycopodium clavatum* and < 500 nm for *Pinus pinaster*). Hence, successful dandelion hollow SEC extraction with removal of the intine layer would yield a complex microstructure featuring significant internal capsule space for potential applications. Of interest, it was also evidenced that the dandelion pollen grains may burst when transferred from a medium of high osmotic pressure to one of low osmotic pressure^19^. Alongside the aforementioned innate robustness of sporopollenin, this special pollen structure and associated properties make dandelion SECs stand out as a potential candidate for the development of drug delivery systems.

Compounding the above advantages of dandelion SECs for drug delivery applications is the general availability and safety of dandelion pollen grains. The two dandelion species, *Taraxacum officinale* and *Taraxacum erythrospermum*, are the most commonly found wild flowers worldwide. They have a long history of being used as medicinal herbs, and have been used as food coloring with the US Food and Drug Administration approving dandelion extracts as “Generally Recognized as Safe”^20–24^. Motivated by these precedents, we decided to investigate the extraction and loading of dandelion SECs.

**Figure 1:**
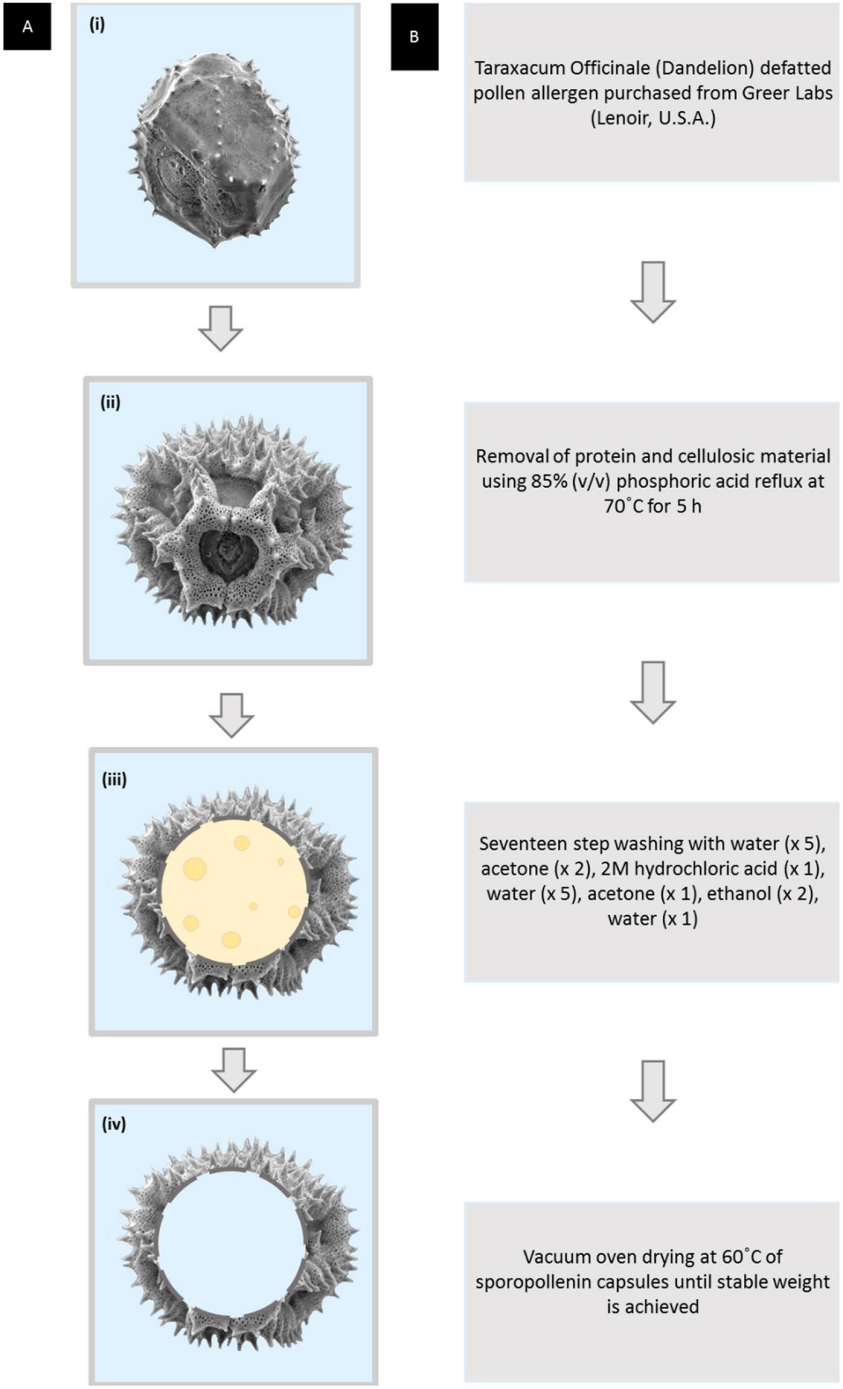
Process of extracting dandelion sporopollenin exine capsules (SECs) from spores. (**A**) Schematic of dandelion spores and SECs, (i) Defatted Taraxacum officinale pollen, (ii, iii) Spores containing sporoplasmic organelles, and (iv) Dandelion SEC after removal of sporoplasmic organelles and other biomolecules. (**B**) Flowchart of process to extract empty clean sporopollenin exine capsules (SECs) from defatted dandelion pollen grains.

Here, dandelion SECs were extracted through acidolysis and alkaline lysis methods in order to retain their sophisticated architecture. The different methods were evaluated by the following criteria: (1) maintenance of complex morphology, (2) remaining protein content analysis, and (3) physical properties of dandelion. Intact protein-free SECs were thus obtained by optimised protocols involving direct application of anhydrous phosphoric acid to pollen samples followed by a series of washing steps (Fig. 1). Finally, bovine serum albumin (BSA) was employed as a model drug to test the loading capability of dandelion SECs.

## Methods

### Materials and methods

*Dandelion* – *Taraxacum Officinale* defatted pollen grains were purchased from Greer Labs (Lenoir, U.S.A.). Phosphoric acid (85% w/v) and hydrochloric acid were purchased from Merck (Singapore). 0.25% trypsin-EDTA and polystyrene-microspheres (50 ± 1 μm) were purchased from Thermo Fisher Scientific (CA, USA). Potassium hydroxide, hydrochloric acid, sodium dodecyl sulfate, and sodium bicarbonate were purchased from Sigma-Aldrich (Singapore). Milli-Q water (Millipore Corp., MA, USA) with resistivity of 18 MΩ cm was used in all the experiments.

### Extraction of dandelion sporopollenin exine capsules (SECs)

Defatted pollen grains were used as the starting material for dandelion SEC extraction. Four extraction protocols were tested: (1) acidolysis, (2) acidolysis followed by trypsin treatment, (3) acidolysis with hydrochloric acid, and (4) alkaline lysis.

For acidolysis, 2 g of defatted dandelion pollen was suspended in 15 ml phosphoric acid at (85% v/v). This was placed in a 50 ml single neck flask fitted with a glass condenser, and refluxed at 70 °C for different time ranges (2.5, 5, 7.5 and 10 h) under gentle stirring (220 rpm). After acidolysis, the sporopollenin capsules were collected by filtration under vacuum and washed in a series of seventeen steps as follows: wash in hot water (5 × 100 mL), hot acetone (2 × 100 mL), hot 2 M hydrochloric acid (1 × 100 mL), hot water (5 × 100 mL), hot acetone (1 × 100 mL), hot ethanol (2 × 100 mL), hot water (1 × 100 mL). The final product was collected by vacuum filtration. Washed SECs were transferred to a clean glass dish, spread to cover the full surface, and air-dried in a fume hood overnight. Drying was then completed in a hot plate oven under vacuum condition for (60 °C, 4 h). Dried SECs were stored in a dry cabinet at room temperature until further characterization.

For acidolysis followed by trypsin treatment, acidolysis as described above was performed with a reflux time of 5 h. Following the subsequent washing steps, SECs were transferred to an Erlenmeyer flask and incubated in 15 mL of 0.25% trypsin-EDTA (37 °C, 1 day). Trypsin was then filtered off, and the SECs washed with water (3 × 100 mL) before incubation in 50 mL of sodium bicarbonate (10 g/L) and sodium dodecyl sulfate (1 g/L) solution (25 °C, 1 day). After incubation, the SECs were washed thoroughly with water, and dried as described above.

For acidolysis with hydrochloric acid, acidolysis as described above was performed with 6 M hydrochloric acid replacing phosphoric acid (85% v/v).

For alkaline lysis, acidolysis as described above was performed with 6% potassium hydroxide replacing phosphoric acid (85% v/v). The solution was then incubated for 3 days at room temperature. Collected SECs were frosted at −20 °C for 1 h, freeze dried for 24 h, and stored in a dry cabinet until further characterization.

### Characterization of surface morphology by scanning electron microscopy (SEM)

FESEM JSM-7600 F (JEOL, Japan) was employed to capture SEM images of dandelion spores and SECs to observe changes in surface structure before and after different stages of chemical with unprocessed and processed capsules. In order to prepare cross-section samples, a small amount of intact SECs or particles was loaded on carbon tape, followed by 30 s of immersion in liquid nitrogen. The particles were then sliced across several times using a steel blade. Samples were freeze-dried overnight or oven dried at 60 °C without vacuum for at least 2 hours to avoid charging effects. Samples were coated with 10 nm thick gold using a JFC-1600 Auto Fine Coater (JEOL, Japan) (18 mA, 100 sec). Images were captured at different magnifications with an acceleration voltage of 5.00 kV. Suitable cracked particles were normally found along the cut lines during SEM session.

### Elemental CHN analysis

To determine protein content in both unprocessed and processed sporopollenin capsules, a calibrated VarioEL III elemental analyzer (Elementar, Hanau, Germany) was used to deliver CHN analyses. The minimum amount of sample needed for CHN analysis was 30 mg. To ensure combustion efficiency, all samples were dried for at least 1 hour at 60 °C beforehand. Triplicate measurements of each sample were taken. The protein content was calculated from percent nitrogen multiplied by the factor of 5.6^21,22^.

### Mass spectrometry analysis

Matrix-assisted laser desorption/ionization - time of flight (MALDI-TOF) mass spectrometry analysis was performed with a Shimadzu Biotech Axima Performance MALDI-TOF system in linear mode, with a power of 100 profiles per run. For sample preparation, 5 mg of pollen or SECs were ground with a mortar and pestle, suspended in 0.5 ml of ethanol, then vortexed for 2 mins. 0.7 μl of the suspension was deposited on a MALDI plate, followed by 0.7 μl of α-Cyano-4-hydroxycinnamic acid (CHCA) matrix solution. This was then dried at room temperature. From each spot, five spectra were recorded at different positions, and data was processed using Gaussian smoothing (100) and baseline correction (100).

### Dynamic image particle analysis (DIPA)

DIPA was performed to determine the effectiveness of processing methods by evaluating the amount of intact sporopollenin obtained, and measuring physical parameters of the sporopollenin (e.g., diameter, circularity, aspect ratio, edge gradient, etc.). FlowCam(Fluid Imaging Technologies, Maine, USA) was employed to capture dynamic image particle, using a 200 µm flow cell (FC-200), 20 × magnification lens (Olympus®, Japan), and FlowCam®. Visual Spreadsheet software version 3.4.11 was used to record data. Before running analysis for the first time, FlowCam® was calibrated using polystyrene microspheres (50 ± 1 µm), with representative data illustrated as a histogram with Gaussian fit.

Before each sample run, the flow cell was cleaned by flushing 1 mL deionized water at a flow rate of 1.0 mL/min. The cleanliness of the flow cell was examined visually before each batch. Unprocessed spores and SECs were vortexed in water and incubated for 10 minutes before filtering with 100 µm mesh to avoid clusters. Sample solutions were manually injected into the flow cell with a pre-run volume of 0.5 mL, and analyzed at a flow rate of 0.1 mL/min, with camera rate at 14 frames/s. This gave a sampling efficiency of approximately 9%. DIPA was stopped when a minimum amount of 15,000 particles was achieved. The process was repeated three times, and data analysis carried out with 300 highly focused particles chosen from the raw data by edge gradient segregation. All values presented take standard deviation into account.

### Confocal laser scanning microscopy analysis (CLSM)

CLSM analysis was carried out to inspect the presence of sporoplasmic cellular organelles and biomolecules in defatted pollens and SECs, and to identify the fluoresceinisothio-cyanate-bovine serum albumin (FITC-BSA)-loaded SECs. A Carl Zeiss LSM710 (Germany) confocal microscope equipped with a Z1 inverted microscope, three spectral reflected/ fluorescence detection channels, and six laser lines (405/458/488/514/561/633 nm) was employed to carry out CLSM analysis.

Samples were mounted on sticky slides (Ibidi, Germany), with a drop of mounting medium (Vectashield®) added before another sticky slide was placed on top. Capturing conditions were set as follows: laser excitation lines 405 nm (6.5%), 488 nm (6%) and 561 nm (6%) with DIC in an EC PlanNeofluar 100 × 1.3 oil objective M27 lens. Fluorescence data from untreated spores and SECs was captured in photomultiplier tubes. The following emission filters were equipped with the photomultiplier tubes: 416–477 nm, 498–550 nm, 572–620 nm. A laser scan speed of 67 seconds per each phase (1024 × 1024: 84.94 µm^2^ sizes) was used in conjunction with plane mode scanning of pixel dwell 12.6 µseconds. The iris was tuned according to sample conditions, and all images were captured within the middle region of the particle. The remaining settings were fixed for all samples. For all samples, at least three images were obtained. Image processing was performed with ZESS 2008 software (ZEISS, Germany).

### Preparation of bovine serum albumin (BSA)-loaded SECs

Bovine serum albumin (BSA)-loaded dandelion SECs were prepared by a vacuum loading method according as previously described, with slightly modification^2,6,17^. Briefly, 150 mg SECs was dispersed in 1.8 mL of 125 mg/mL BSA solution by vortexing for 10 min. The suspension was then transferred to a freeze dryer with 0.008 mbar vacuum for 4 h. The samples were collected, washed twice by using 2 mL deionized water, and centrifuged at 4700 rpm to remove unloaded BSA. Finally, the BSA-loaded SECs were freeze-dried for 12 h. For control groups, defatted pollen and SECs were similarly prepared in the absence of BSA.

### Bovine Serum Albumin (BSA)-loading content and encapsulation efficiency

5 mg of BSA-loaded dandelion SECs were suspended in 1.4 mL of phosphate buffer saline (PBS), vortexed for 5 min, and probe sonicated for 3 cycles of 10 s at 40% amplitude. The solution was filtered to collect the extracted BSA using a 0.45 μm polyethersulfone (PES) syringe filter (Agilent, CA, USA). BSA content was assessed by UV absorption at 280 nm (Boeco-S220, Germany) using corresponding control group as a blank. BSA-loading content and encapsulation efficiency of the BSA-loaded SECs were calculated using the following equations:

$$\begin{array}{c}{\rm{BSA}}\,{\rm{loading}}\,{\rm{content}}( \% )={\rm{amount}}\,{\rm{of}}\,{\rm{BSA}}\,{\rm{in}}\,\mathrm{BSA}-\mathrm{loaded}\,\mathrm{SECs}/\mathrm{amount}\,{\rm{of}}\,\mathrm{BSA}-\mathrm{loaded}\\ \,\,\,\,\,\,\,\,\,\,\,{\rm{SECs}}\times {\rm{100}}\end{array}$$

$$\begin{array}{c}{\rm{Encapsulation}}\,{\rm{efficiency}}( \% )={\rm{practical}}\,{\rm{BSA}}\,{\rm{loading}}\,\mathrm{content}/\mathrm{Theoretical}\,{\rm{BSA}}\,{\rm{loading}}\\ \,\,\,\,\,\,\,\,\,\,\,\,\,\,{\rm{content}}\times 100.\end{array}$$

### Statistical analysis

Statistical comparisons were performed using two-tailed t-tests with P < 0.05 considered as statistically significant. Data presented are the mean values ± standard deviation of three separate measurements.

## Results and Discussion

### Extraction of dandelion SECs using different methods

Following our previous work on the extraction of sunflower (*Healianthus annuus*)^17^, moss (*L*.*clavatum*)^23^, corn (*Zea mays*)^18^ and pine (*Pine taeda*)^24^, defatted dandelion pollens were subjected to phosphoric acid reflux, hydrochloride acid reflux, and alkaline lysis methods. Briefly, based on morphological analysis of SECs obtained by the three strategies, we chose phosphoric acid reflux as the most promising method. The optimized process consists of phosphoric acid (85% v/v) reflux at 70 °C for 5 hours, followed by a series of washing steps (Fig. 1). Compared to the defatted pollen, the final products not only demonstrated removal of protein, lipid, cellulose, and other extraneous materials – but also maintenance of the intricate microridge structural integrity. It is seen that high quality dandelion SECs could therefore be obtained using our protocol, with our hollow and uniform SECs meeting a standard for microencapsulation and potential drug delivery applications.

### Micrometric analysis of dandelion SECs

**Figure 2:**
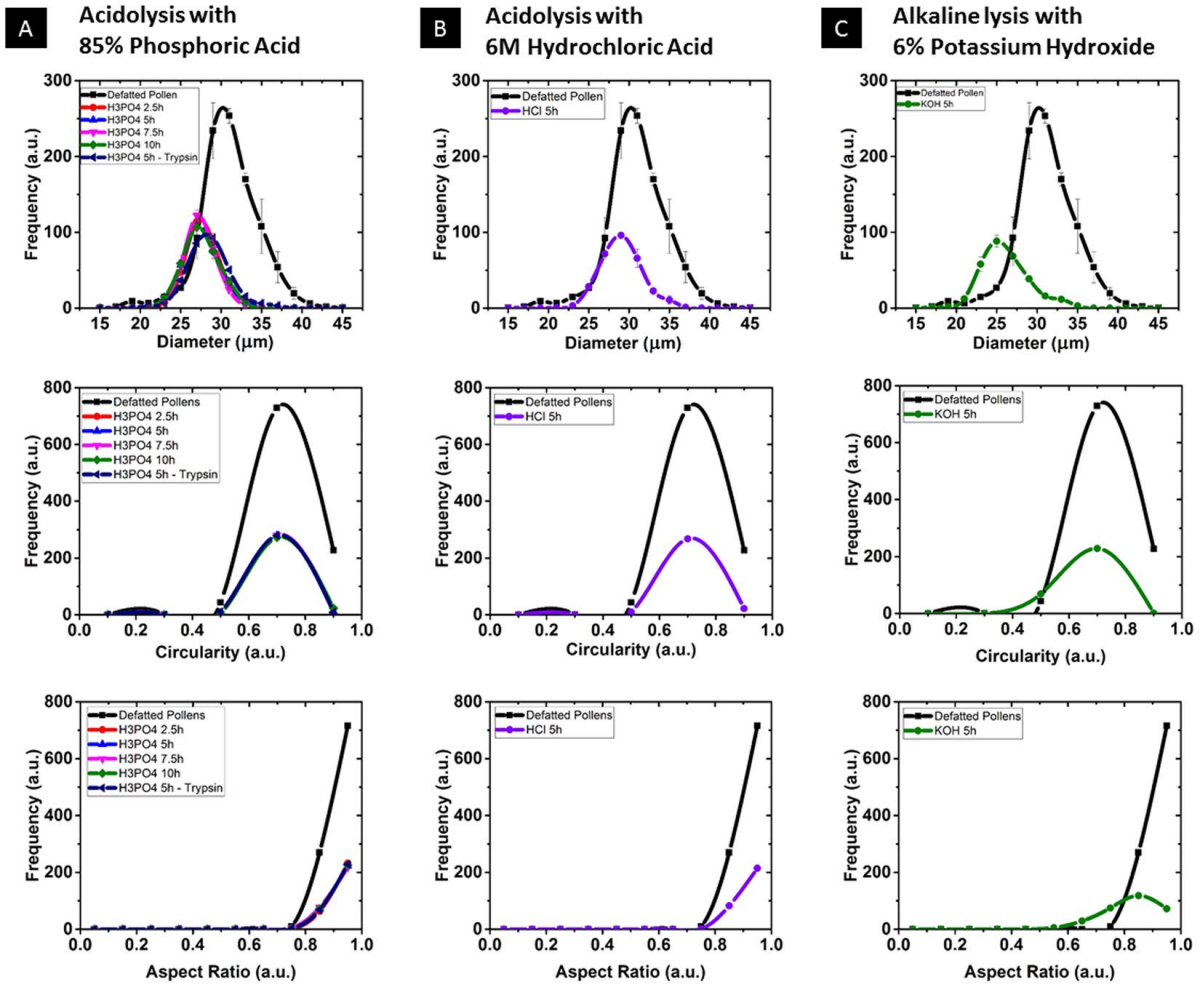
Characterization of sporopollenin exine capsules (SECs) in various treatment conditions using dynamic imaging particle analysis (DIPA). Micromeritic properties of SECs are illustrated as column (**A**) Acid treatment using 85% (v/v) phosphoric acid at 70 °C for different time ranges, column (**B**) Acid treatment using 6 M hydrochloric acid at 70 °C for 5 hours and column (**C**) Alkaline lysis using 6% Potassium Hydroxide at 70 °C for 5 hours. Data was obtained by the spline curve fitting of histogram from 300 well-focused images after triplicate measurements (n = 3) and plotted as graphs of diameter, circularity, and aspect.

To examine particle size and monodispersity, high-throughput screening of SECs and unprocessed pollen using dynamic imaging particle analysis (DIPA) was carried out. The pollen/SEC size and morphology was characterized by their distribution of diameter, circularity, and aspect ratio (Table 1 and Fig. 2). Here, aspect ratio is defined as the ratio between the minor axis and major axis of the object image fitting ellipse^25^.

**Table 1:** Summary of the micrometric parameters of dandelion SECs under different treatments.

Treatment	Diameter (µm)	Circularity	Aspect ratio	Polydispersity index
Defatted Pollen	29.28 ± 2.70	0.99 ± 0.00	0.92 ± 0.04	0.009 ± 0.004
85% H_3_PO_4_ 2.5 h	27.80 ± 2.12	0.99 ± 0.00	0.93 ± 0.04	0.006 ± 7.9E-5
85% H_3_PO_4_ 5 h	27.73 ± 2.35	0.99 ± 0.00	0.92 ± 0.04	0.007 ± 0.001
85% H_3_PO_4_ 7.5 h	27.32 ± 1.98	0.99 ± 0.01	0.92 ± 0.04	0.005 ± 3.3E-4
85% H_3_PO_4_ 10 h	27.46 ± 2.63	0.99 ± 0.00	0.92 ± 0.04	0.007 ± 3.2E-4
85% H_3_PO_4_ 5h-Trypsin	28.69 ± 2.71	0.99 ± 0.00	0.92 ± 0.04	0.009 ± 0.001
6 M HCl 5 h	29.04 ± 2.52	0.99 ± 0.00	0.92 ± 0.04	0.008 ± 8E-4
6% KOH 5 h	26.09 ± 2.97	0.95 ± 0.04	0.82 ± 0.09	0.013 ± 0.001

The data sets are the same with that in Fig. 2 and shown as means ±  standard deviation (n = 3).

**Figure 3:**
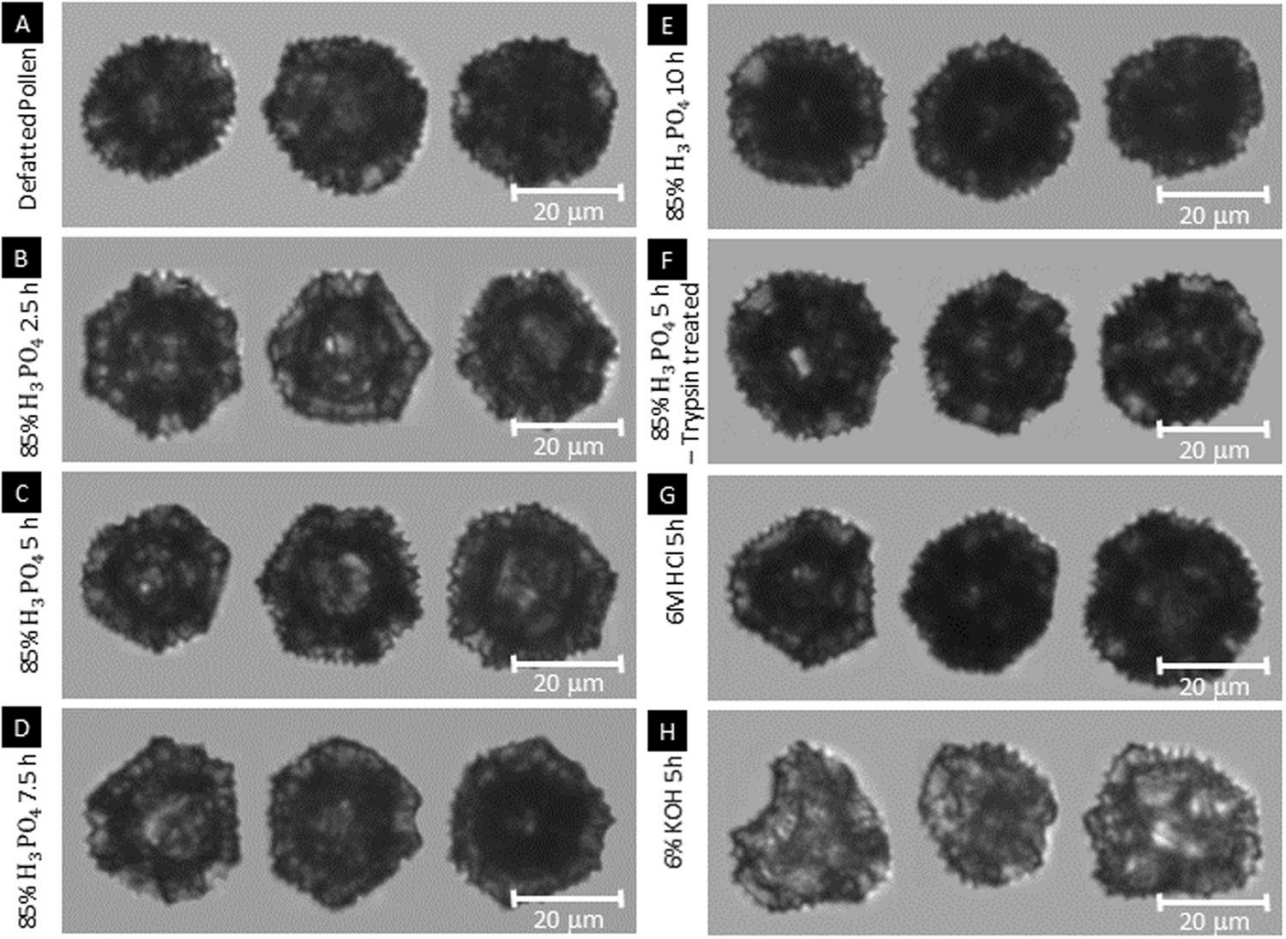
Sporopollenin exine capsule (SEC) based on dynamic imaging particle analysis (DIPA) (**A**) Representative DIPA images of particles in the size range of 18–39 µm from defatted dandelion pollen (**B–H**) Representative DIPA images of particles in the size range of 18–39 µm from processed SECs.

DIPA images of untreated dandelion pollen display a distinctive hexagonal microstructure with surface ornamentation covered with spikes, with spike integrity directly affecting size distribution measurements. After the extraction through acidolysis with both phosphoric and hydrochloric acid, most of the dandelion SECs (>97%) retained their intrinsic microstructure without any alternation of sophisticated nanoscale features, including spikes (Fig. 3 and Table 2). Size distribution of SECs indicate that the phosphoric acid extraction produces SECs of high monodispersity and similar structural ornamentation as defatted pollens. Furthermore, upon acidolysis with phosphoric acid, the Equivalent Spherical Diameter (ESD) reduced from 29.28 ± 2.70 µm to 27.46 ± 2.26 µm in the case of defatted dandelion pollen (Table 1 and Fig. 2). Meanwhile, the low broken particles ratio indicate that the dandelion SECs possess robust mechanical performance in the phosphoric acid and/or hydrochloric acid treatment Tables 3 and 4.

**Table 2:** Ratio of intact and broken defatted pollen and dandelion sporopollenin exine capsules (SECs).

Process	Broken Particles (%)
Defatted Pollen	1.2 ± 0.2
85% H_3_PO_4_ 2.5 h	1.8 ± 0.2
85% H_3_PO_4_ 5 h	2.2 ± 0.5
85% H_3_PO_4_ 7.5 h	2.4 ± 0.7
85% H_3_PO_4_ 10 h	3.0 ± 0.6
85% H_3_PO_4_ 5 h - Trypsin	2.3 ± 0.3
6 M HCl 5 h	1.4 ± 0.5
6% KOH 5 h	95.7 ± 0.9

Broken particles were counted as the deformed pollens among 300 chosen intact particles captured using DIPA FlowCam®. Data represented is an average of triplicate measurements with standard deviation (n = 3).

**Table 3:** CHN composition of defatted dandelion pollen and dandelion sporopollenin exine capsules (SECs).

Process conditions	Carbon (%)	Hydrogen (%)	Nitrogen (%)
Defatted Pollen	43.6 ± 0.4	7.7 ± 0.1	2.3 ± 0.0
85% H_3_PO_4_ 2.5 h	62.7 ± 0.1	7.6 ± 0.0	0.9 ± 0.0
85% H_3_PO_4_ 5 h	62.7 ± 0.9	7.7 ± 0.2	0.9 ± 0.0
85% H_3_PO_4_ 7.5 h	63.1 ± 0.3	8.0 ± 0.0	1.0 ± 0.0
85% H_3_PO_4_ 10 h	63.1 ± 0.2	7.9 ± 0.0	1.0 ± 0.0
85% H_3_PO_4_ 5 h - Trypsin	62.9 ± 0.1	7.7 ± 0.0	0.8 ± 0.0

The CHN content was reported as average values with standard deviation from triplicate results of CHN analysis.

**Table 4:** Loading content and encapsulation efficiency of defatted dandelion pollens and dandelion SECs.

Material	Theoretical loading content (%)	Loading content (%)	Encapsulation efficiency (%)
Defatted dandelion pollens	60	12.65 ± 1.44	21.10 ± 2.4
Dandelion SECs	60	32.23 ± 0.33	53.72 ± 0.54

Shown are means ± SD from triplicate.

However, for the SECs produced by alkaline lysis, monodispersity is lower than that produced by acidolysis, and most of them appeared collapsed or fractured (about 96%) (Fig. 3 and Table 2). The damaging effects on particle integrity were further evidenced by the corresponding trend in aspect ratio, which decreased from 0.92 ± 0.04 to 0.82 ± 0.09 before and after alkaline lysis, respectively, along with an increased polydispersity index (from 0.009 ± 0.004 to 0.013 ± 0.001) (Table 1).

### Morphology of defatted dandelion pollen and dandelion SECs

To investigate the morphological character of defatted dandelion pollen and the SECs, scanning electron microscopy (SEM) was employed to examine their surface and cross-sectional topography. Defatted dandelion pollen has a distinctive microstructure (spheroidal with a triangular and long spines) with a uniform size distribution (about 23–27 µm). This is smaller than that measured by DIPA as samples are dried and placed in vacuum in SEM, with expected particle shrinkage occurring. Furthermore, for defatted dandelion pollen, debris filling the lacunae (openings of the exine) is observed. This is likely to be residual pollenkit, the largely lipid-composed material that surrounds pollen grains which is known to remain even after the defatting process^26^.

**Figure 4:**
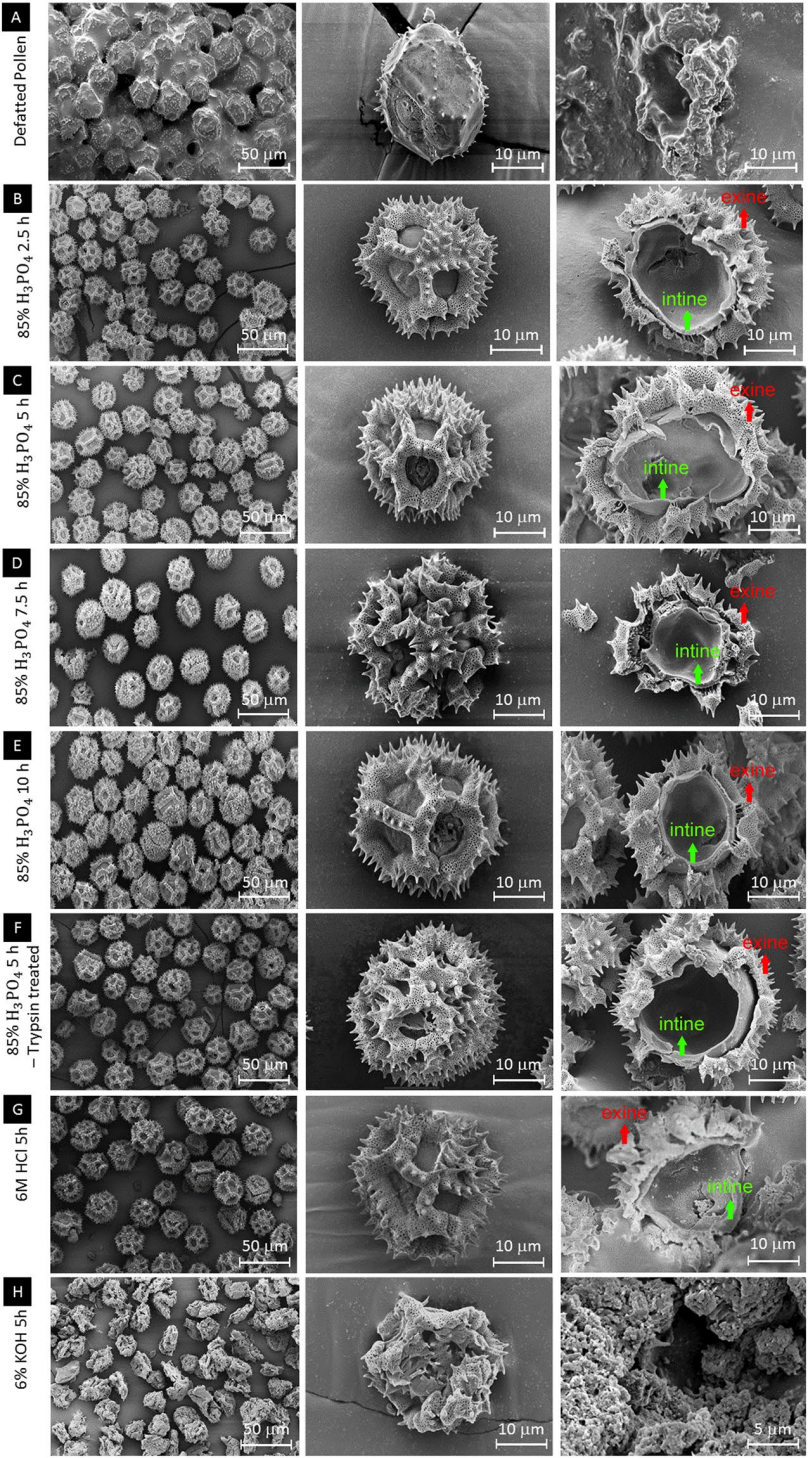
Evaluation of sporopollenin exine capsules (SECs) after prolonged acidolysis by scanning electron microscopy. (**A**) Defatted spores at different magnifications are clustered and covered with biomolecules; the cross section indicates the presence of sporoplasmic biomolecules. (**B–E**) SECs after acidolysis with 85% Phosphoric Acid for 2.5 h, 5 h, 7.5 h and 10 h respectively at different magnifications, indicating intact, well defined microstructure with empty inner cavity. (**F**) SECs treated with 0.25% Trypsin – EDTA after 5 h acidolysis with 85% (v/v) Phosphoric Acid at different magnifications show intact and clean capsules. (**G**) SECs after 5 h acidolysis using 6 M Hydrochloric Acid at different magnitudes indicating intact capsules with the cross section revealing residual proteinaceous debris and the cellulosic intine layer. (**H**) SECs after alkaline lysis using 6% Potassium Hydroxide at different magnitudes were clustered together and severely damaged with the presence of debris and broken particles.

After acidolysis processing, the complex and porous SECs are imaged, with elaborate surface features in the form of ~700 nm-thick echinate spikes and ~3.5 µm-wide lophate porous ridges observed (Fig. 4). In more detail, after refluxing for 2.5 h to 10 h, there are no modification observed in external SEC morphology and microstructures. Moreover, for all of the 2.5 h to 10 h phosphoric acid treated SECs, the inner cavity appeared to be clean, empty and smooth. Prolonged acidolysis for 7.5 h and 10 h did not convey significant modifications in term of internal and external morphology and microstructure (Fig. 4). Overall, it is seen that acidolysis using phosphoric acid (85% v/v) with extensive water and solvent washing resulted in clean, intact, and monodisperse SECs

In comparison, acidolysis with trypsin treatment also showed no change in the architecture of the dandelion SECs. However, with acidolysis using 6 M HCl, although surface structure was retained, significant amounts of residual debris remained on the pollen surface and the inner cavity (Fig. 4). Finally, alkaline lysis using KOH severely damaged the spiky microstructure of the SECs, as the loss of their unique microstructure was observed. These results indicate that acidolysis using phosphoric acid (85% v/v) with extensive water and solvent washing is a practical method to prepare clean and intact dandelion SECs.

### Protein removal from the dandelion pollen

**Figure 5:**
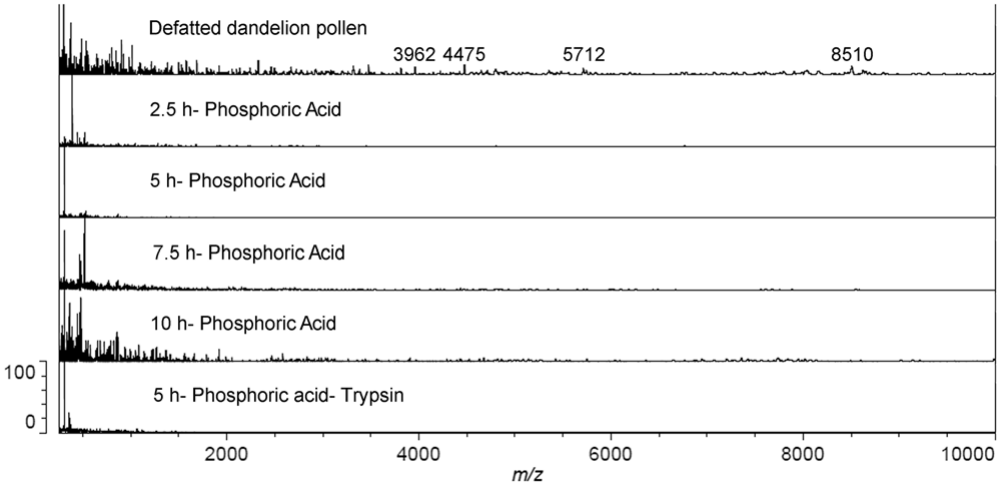
MALDI-TOF spectrum for defatted dandelion pollens and SECs. All the sample was prepared as described in “Material and method section”. The relative intensity was shown on the Y-axis. The mass to charge ration (m/z) is demonstrated on the X-axis in Dalton. Data is presented as representative of triplicate measurements.

Based on the knowledge that protoplasmic components mainly include fats, genetic material and proteins^27,28^, we used MALDI-TOF to monitor the macromolecular protein degradation in pollen after acidolysis with phosphoric acid, and acidolysis with trypsin treatment (Fig. 5). The results showed that with unprocessed pollen, multiple peaks can be found in the range of 4000 m/z to 9000 m/z. However, these peaks were absent in mass spectroscopy of all processed SECs (Fig. 5), suggesting that most of the protoplasmic biomacromolecules degraded after acidolysis.

**Figure 6:**
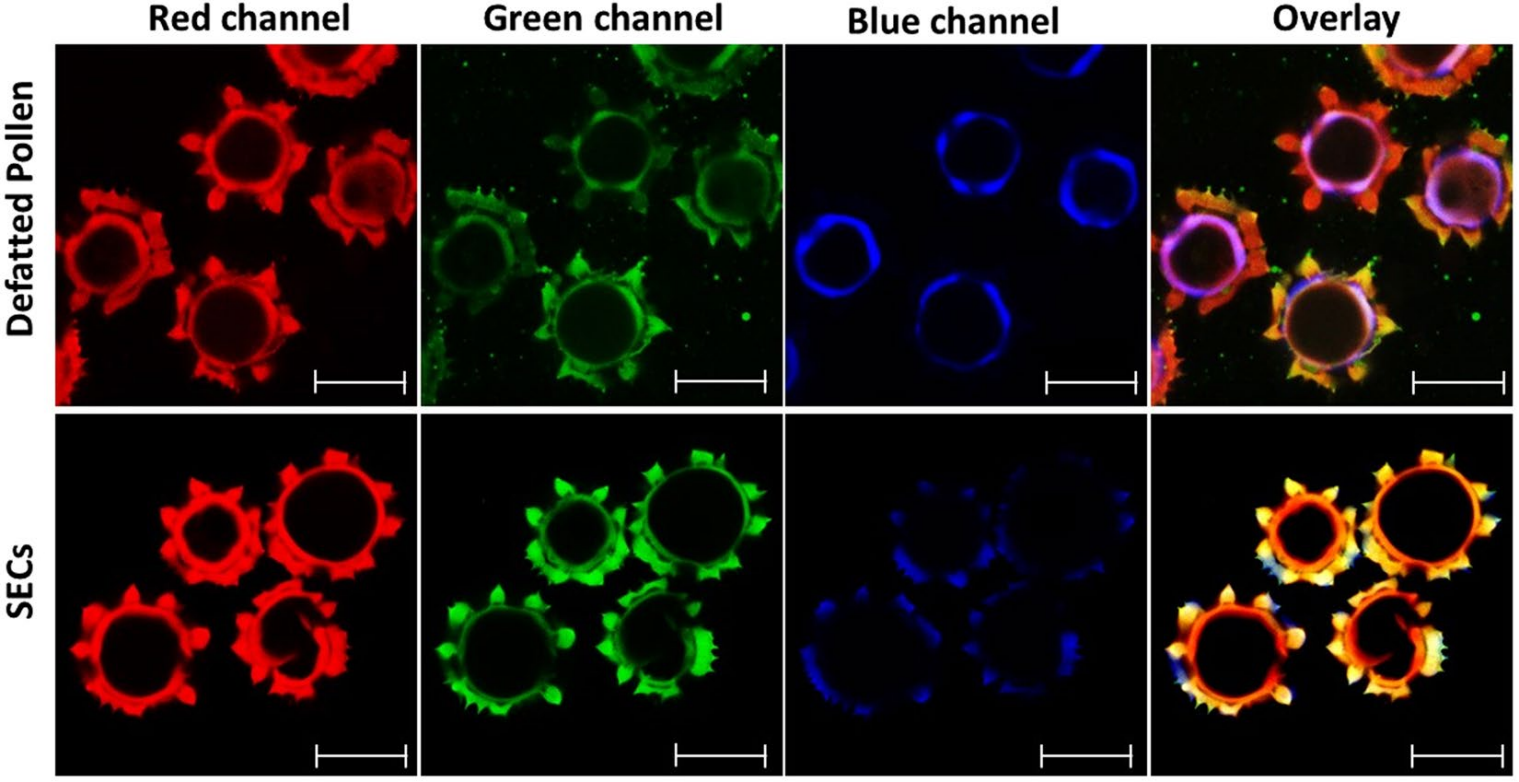
Confocal laser scanning microscopy (CLSM) analysis of sporopollenin exine capsules (SECs) before and after treatment. In the first row, CLSM images of defatted dandelion spores are presented with autofluorescence effect owing to the appearance of sporoplasmic cellular organelles and biomolecules. The second row shows intact SECs after 5 h acidolysis-only treatment with a clean and large inner cavity. Some broken particles are recorded. (Scale bars are 20 µm).

Furthermore, confocal laser scanning microscopy analysis was carried out to further demonstrate pollen removal after the extraction process. It is common knowledge that pollen grains experience auto-fluorescence due to their inner constituents^29^. Confocal microscope images of defatted dandelion spores before and after processing are shown in Fig. 6. It is evident from the first row that there is strong autofluorescence inside the cavity with unprocessed defatted dandelion pollen. However, no autofluorescence is identified inside the SECs after acidolysis with phosphoric acid, indicating removal of the inner cavity sporoplasm. The data from CLSM evaluation also supports that the acidolysis using phosphoric acid produces high yield of complete capsules even though some broken particles can be observed.

**Figure 7:**
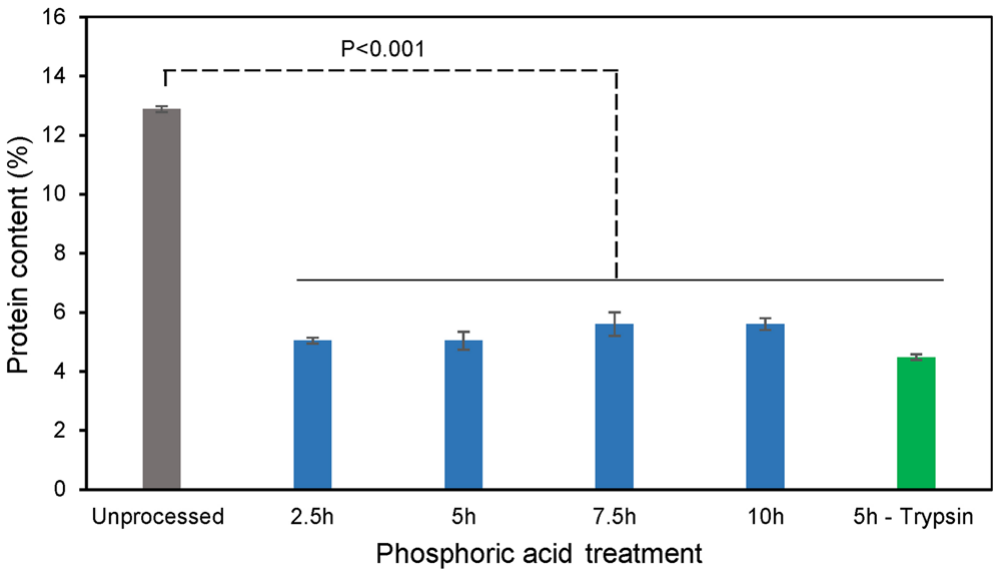
Protein content in dandelion sporopollenin exine capsules (SECs) in different treatment conditions. The protein content is obtained from CHN elemental analysis and indicates the level of sporoplasm constituents in defatted spores and processed SECs. Data displayed is the average of triplicate measurements with standard deviation.

Considering that proteins are the only major component of pollen cytoplasm that contain nitrogen^4^, CHN element analysis was performed to further verify the protein degradation. Results of CHN analysis indicate that defatted dandelion contains $\sim$12.88 wt% of proteins (Fig. 7), in agreement with the previous literature^22^. Meanwhile, acidolysis with phosphoric acid resulted in the largest overall reduction in protein, to less than 6%, even after only 2.5 h of treatment. Beyond 2.5 h, no significant reduction in nitrogen content was observed. Therefore, acidolysis at 70 °C for 2.5 hours with phosphoric acid (85% v/v) was seen to efficiently cause proteinaceous nitrogen removal.

On a final note, although MALDI-TOF analysis indicated that there was a negligible amount of protein present in the SECs, peaks were observed indicating a considerable amount of nitrogen, pointing to the presence of other nitrogen-containing compounds in the dandelion SEC matrix. One possible reason for this is the formation of stable nitrogen-containing compounds produced during the SEC extraction process.

### Microencapsulation of BSA into dandelion SECs

To verify that the dandelion SECs can be used as a drug delivery vehicle, BSA was chosen as a drug model and loaded into the dandelion SECs by the vacuum loading method previously described. Vacuum loading of BSA into defatted dandelion pollens was also performed as a control. UV-Vis analysis of the dandelion SECs showed a 2.55-fold and 3.25-fold higher loading content and encapsulation efficiency than defatted dandelion pollen. The high BSA loading content (32.23 ± 0.33%) and encapsulation efficiency (53.72 ± 0.54%) strongly suggest that the dandelion SECs have significant potential as a drug carrier.

**Figure 8:**
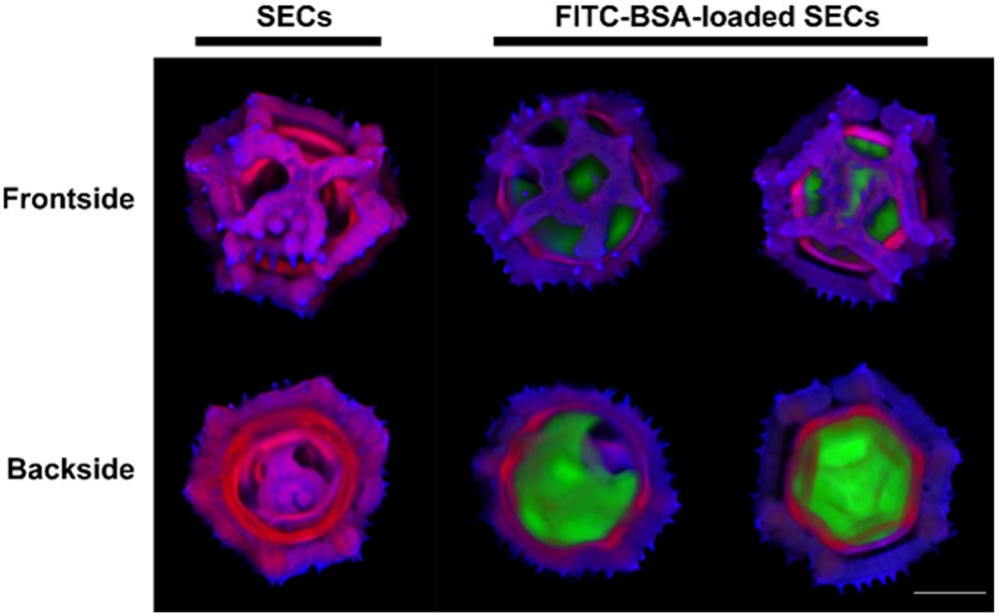
3D CLSM images of dandelion sporopollenin exine capsules (SECs) before and after FITC-BSA loading. 3D images were constructed by the CLSM z-stack function. The frontside refers to the bottom of the selected SEC and the backside is a cross-sectional image which is the layer about 21 µm away from the bottom layer of the SECs. (Scale bars are 10 µm).

To further evaluate the loading process, FITC-BSA was loaded into SECs using the same loading procedure. The location of FITC-BSA in SECs was then observed using confocal laser scanning microscopy (CLSM). The microscope was first calibrated to produce no visible SEC autofluorescence emissions in the green spectrum. By using the CLSM z-stack imaging function, the frontside (the bottom of the SECs) and backside (about 21 µm count from the bottom of the SECs) of the SECs and FITC-BSA-loaded SECs were 3D imaged (Fig. 8). Captured images suggested that clean, intact SECs were obtained with no green fluorescence, and that BSA was successfully encapsulated into SECs with high loading content and no BSA residues on the surface of SECs.

Interestingly, compared with other pollens species^17,24^, the defatted dandelion pollen showed a considerably higher loading content (12.65 ± 1.44%). This may be attributed to its large inner cavity. Future work will investigate the potential of these large inner cavities for microencapsulation.

## Conclusion

Sporopollenin has significant potential in a variety of applications – from drug delivery, to cosmetics, or even the food industry. Dandelion SECs are of particular interest based on their complex architecture and large internal cavities. To prepare these SECs for such future applications, it is crucial to develop a simple and non-toxic process to isolate intact, clean SECs without damaging their intrinsic architecture. Based on the results obtained throughout the course of this work, highly monodisperse, intact and clean dandelion SECs may be obtained through acidolysis using phosphoric acid at an elevated temperature. Data obtained from DIPA and SEM imaging, demonstrate the intact and monodisperse nature of extracted dandelion SECs; whereas data obtained from SEM, MALDI-TOF, CHN elemental analysis and CLSM demonstrate the removal of extraneous materials from the sporopollenin. Furthermore, BSA was successfully loaded into the SECs with high loading content and encapsulation efficiency, supporting the idea that dandelion SECs could be a promising shell material for microencapsulation and drug delivery. In summary, the results of this work should help build a foundation for large-scale production of SECs for potential applications and contribute to the database of new SECs extracted from a wide variety of plant species.
